# COVID-19 infection during pregnancy: A retrospective study in a city in the Southeastern region of Turkey

**DOI:** 10.15537/smj.2023.44.3.20220729

**Published:** 2023-03

**Authors:** Fethiye Akgül, Can Tüzer, Yusuf Arslan, Bünyamin Sevim

**Affiliations:** *From the Department of Infectious Diseases and Clinical Microbiology (Akgül, Arslan); from the Department of Allergy and Immunology (Tüzer), Batman Training and Research Hospital, and from the TR Ministry of Health (Sevim), Batman Provincial Health Directorate, Batman, Turkey.*

**Keywords:** COVID-19, pregnancy, SARS-CoV-2, trimester, COVID-19 vaccination

## Abstract

**Objectives:**

To evaluate the maternal and fetal clinical outcomes in SARS-CoV-2 infected pregnant women during the whole period of the pandemic in a single province in the southeast of Turkey.

**Methods:**

This retrospective study included patients who were screened from the medical registration system and found to be infected with SARS-CoV-2 virus during pregnancy. The demographic, clinical, laboratory, and radiological features of all the patients were obtained and compared between groups classified as severe-critical and mild-moderate disease severity.

**Results:**

The mean age of all the cases was 29.0±5.3 years in the mild-moderate cases, and 30.1±5.5 years in the severe-critical cases. The rates of 3rd trimester, cesarean and premature birth, high body mass index (BMI), symptoms of cough and dyspnea, the presence of comorbidities, and hypothyroidism were significantly higher in the severe-critical cases than in the mild-moderate group. In the univariate analyses, BMI, dyspnea, cough, maternal complication rate, the neutrophil/lymphocyte ratio, the values of white blood cells, procalcitonin, high-sensitive C-reactive protein, D-dimer, ferritin, aspartate aminotransferase, and alanine aminotransferase were detected as significant risk factors. In the multivariate analysis, only procalcitonin was a significant factor.

**Conclusion:**

In the 3rd trimester of pregnancy, obesity and hypothyroidism were found to be risk factors for severe-critical cases of COVID-19 infection, and the clinical course was more severe with a higher rate of mortality in the recent period of the pandemic.

**S**evere acute respiratory syndrome-related coronavirus-2 (SARS-CoV-2) virus was determined as the agent of COVID-19, which rapidly progressed to become a global pandemic. Since the first outbreak in December 2019, it has not been fully clarified who has been most severely affected by this virus. The clinical presentation ranges from asymptomatic disease to multi-organ failure. The elderly and individuals with immunodeficiency are known to be prone to have a more severe disease.^1,2^ Pregnancy is also an immunosupressive process that makes women susceptible to viral infections. As a result of immunosupression and the substantial changes in the cardiorespiratory system, pregrant women are more vulnerable to a severe disease course during respiratory viral infections.^3^ During the H1N1 pandemic in 2009, pregnant subjects comprised 1% of H1N1-infected patients, and 5% of H1N1-infected patients with mortality were reported to be pregnant women.^4^ Severe acute respiratory syndrome-related coronavirus-2 and Middle East respiratory syndrome coronavirus infections during pregnancy have been assumed to be responsible for severe clinical outcomes such as admission to intensive care units (ICUs), endotracheal intubation, renal failure, and even death.^5,6^ At the beginning of the COVID-19 pandemic, there were concerns regarding pregnant women because of the harmful effects of viral infections such as pneumonia. Nevertheless, pregnant women with COVID-19 infection have not been reported to have experienced more harmful effects of the virus and vertical transmission has not been shown precisely to date.^7,8^ More recently, an increase in admissions to ICUs and mortality rates has come to the fore because of new variants of the virus. However, there are currently a limited number of studies on this issue.

The aim of this study was to evaluate the maternal and fetal clinical outcomes in SARS-CoV-2 infected pregnant women, including the differences between patients who became infected in each of the 3 trimesters, from March 2020 until September 2021 in a single province in southeast Turkey.

## Methods

The patients were screened retrospectively from the medical records in the public health management system and hospital management system, which are both registration systems in Turkey. The study included pregnant women determined with COVID-19 positivity who presented between March 2020 and September 2021. The patients included were those with confirmed COVID-19 positivity during pregnancy, who provided written informed consent for participation in the study via messenger applications on mobile phones. Patients were excluded from the study if they were diagnosed with COVID-19 before or after pregnancy, if they could not be contacted by telephone, or if they did not give informed consent.

The demographic, clinical, laboratory, and radiological features of all the patients were obtained from hospital records. The course of the pregnancy, treatment options, and the health status of the newborns were also obtained from hospital records and assessed in detail. The patients were categorised according to body mass index (BMI) as underweight (<18.5 kg/m^2^), normal (18.5-24.9 kg/m^2^), pre-obesity (25-29.9 kg/m^2^), obesity class I (30-34.9 kg/m^2^), obesity class II (35-39.9 kg/m^2^), and obesity class III (>40 kg/m^2^).^9^

All the patients included in the study were assessed according to the clinical findings on first admission to hospital and 2 groups were formed as patients with mild-moderate severity of COVID-19, and patients with severe-critical COVID-19. These 2 groups were compared in respect of the relevant variables examined in the study. Coronavirus disease-19 severity was assessed according to the following criteria: I) mild (no pneumonia); II) moderate (pneumonia present with respiratory symptoms but no accompanying hypoxia; III) severe (respiratory rate of ≥30/min, oxygen saturation of <94%, PaO2/FiO2<300, pulmonary infiltration of >50% [involvement of >50% of the total lung parenchyma area as pulmonary infiltration or ground-glass opacities on thorax computed tomography {CT}] and dyspnea; and IV) critical (respiratory failure, septic shock, and multi-organ failure requiring mechanical ventilation).^10^

The demographic, clinical and laboratory findings, treatment options, and maternal and neonatal outcomes were compared between the severe-critical and mild-moderate cases. The demographic and clinical outcomes of the pregnant women with confirmed COVID-19 infection in a single province of Turkey during the whole period of the pandemic were evaluated in detail.

Approval for the study was granted by the Ethics Committee of Batman Training and Research Hospital, Batman, Turkey, with decision number 280, on 8th September 2021. The necessary permission for the study was obtained from the Turkish Health Ministry and the Provincial Health Department in Batman, Turkey.

### Statistical analysis

Descriptive statistics were used to evaluate the demographic and clinical characteristics. Quantitative variables not showing normal distribution were analyzed using the Mann-Whitney-U test and in the comparisons of categorical variables, the Chi-square test was applied. Multivariate analysis with binary logistic regression analysis was carried out for the associated factors obtained from univariate analysis with binary logistic regression analysis. The optimal cut-off threshold was determined according to receiver operating characteristic curve (ROC) analysis. All data analyses were carried out with the Statistical Package for the Social Sciences, version 15.0 (SPSS Inc, Chicago, IL, USA). A *p*-value of<0.05 was considered significant.

## Results

Evaluation was carried out of a total of 291 pregnant women infected with SARS-CoV-2 virus. During the defined period, the total number of COVID-19-positive patients was 58,811, of which 1178 (2%) were pregnant women. The population in Batman, Turkey, is 620,000, and the positivity rate of COVID-19 in the province was estimated to be 9.5% between March 2020 and September 2021. In the same time period, the number of pregnant women was 19,800.

**Figure 1:**
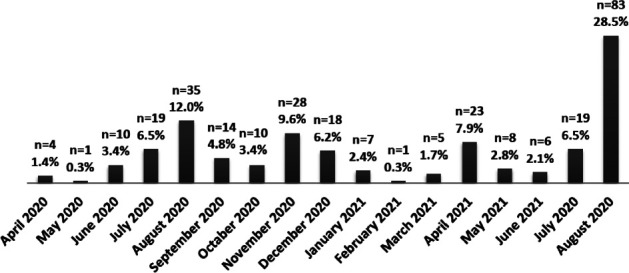
- Distribution of our cases by month.

When the distribution of the patients diagnosed with COVID-19 infection was examined by months, the majority were seen in August 2021 (Figure 1). The mean age was determined as 29.0±5.3 years for all the cases, 29.0±5.3 years for the mild-moderate cases, and 30.1±5.5 years for the severe-critical cases (*p*>0.05).

A total of 4 pregnant cases developed mortality. The mean age of these cases was 34 years, and all were a singleton pregnancy. The initial symptoms were cough, fatigue, and dyspnea in all 4 subjects. One subject had gestational diabetes mellitus and one hypothyroidism. None of them had received a COVID-19 vaccination. All 4 cases were in the third trimester of pregnancy and the type of delivery was cesarean section. All the newborns were healthy.

Body mass index measurements (*p*=0.014), the presence of cough (*p*<0.001), dyspnea (*p*<0.001), pre-existing comorbidities (*p*=0.041), and hypothyroidism (*p*=0.041) were significantly higher in the severe-critical cases than in the mild-moderate cases (Table 1). The rates of third trimester (*p*=0.013), hospitalization (*p*<0.001), mortality (*p*<0.001), chest X-ray (*p*<0.001), and CT screening (*p*<0.001) were significantly higher in the severe-critical cases than in mild-moderate cases (Table 2). The mild-moderate cases did not receive any COVID-19 treatment. In the severe-critical group, treatment was administered of ritonavir-lopinavir (2 patients), favipravir (9 patients), plaquenil (9 patients), systemic steroid (5 patients), immune plasma (one patient), intravenous immunoglobulin (2 patients), and anti-IL6 (tocizulumab) (one patient; Table 2). The rates of cesarean sections (*p*=0.004), premature births (*p*<0.001), and COVID-19 testing of newborns with reverse transcriptase-polymerase chain reaction (*p*=0.001), length of hospital stay of the newborns (*p*<0.001), and maternal complications (cough, dyspnea, fatigue, headache, muscle pain, loss of taste and smell, and other; *p*=0.0024) were also higher in severe-critical patients than in the mild-moderate group.

**Table 1:** - Demographic characteristics of the pregnant women in the study.

Variables	Total	Disease severity		*P*-values
		Mild-moderate	Severe-critical	
Age, mean±SD (min-max)	29.0±5.3 (18-42)	29.0±5.3 (18-42)	30.1±5.5 (23-38)	0.566
***BMI (kg/m^2^)***				
Underweight (<18.5)	12 (4.1)	12 (4.3)	0 (0.0)	0.014
Normal (18.5-24.9)	131 (45.0)	130 (46.1)	1 (11.1)	
Pre-obesity (25-29.9)	101 (34.7)	98 (34.8)	3 (33.3)	
class I obesity (30-34.9)	34 (11.7)	29 (10.3)	5 (55.6)	
class II obesity (35-39.9)	12 (4.1)	12 (4.3)	0 (0.0)	
Class III obesity (>40)	1 (0.3)	1 (0.4)	0 (0.0)	
Number of pregnancies, mean±SD (min-max)	2.7±1.6 (1-11)	2.7±1.6 (1-11)	3.0±1.7 (2-5)	0.238
***Pregnancy status***				
Sıngle	288 (99.0)	279 (98.9)	9 (100)	1.000
Twins	3 (1.0)	3 (1.1)	0 (0.0)	
Smoker	20 (6.9)	18 (6.4)	2 (22.2)	0.121
***Complaints***				
Absent	6 (2.1)	6 (2.1)	0 (0.0)	1.000
Present	285 (97.9)	276 (97.9)	9 (100)	
Fever	20 (6.9)	18 (6.4)	2 (22.2)	0.121
Weakness	216 (74.2)	209 (74.1)	7 (77.8)	1.000
Nausea-vomiting	21 (7.2)	21 (7.4)	0 (0.0)	1.000
Taste-smell loss	93 (32.0)	92 (32.6)	1 (11.1)	0.280
Shortness of breath	50 (17.2)	42 (14.9)	8 (88.9)	<0.001
Headache	82 (28.2)	80 (28.4)	2 (22.2)	1.000
Diarrhea	9 (3.1)	9 (3.2)	0 (0.0)	1.000
Cough	37 (12.7)	29 (10.3)	8 (88.9)	<0.001
***Comorbidities***				
Absent	244 (83.8)	239 (84.8)	5 (55.6)	0.041
Present	47 (16.2)	43 (15.2)	4 (44.4)	
CAD	3 (1.0)	2 (0.7)	1 (11.1)	0.090
HT	2 (0.7)	2 (0.7)	0 (0.0)	1.000
DM	2 (0.7)	2 (0.7)	0 (0.0)	1.000
Asthma	16 (5.5)	16 (5.7)	0 (0.0)	1.000
Rheumatological disease	2 (0.7)	2 (0.7)	0 (0.0)	1.000
Gestational DM	6 (2.1)	5 (1.8)	1 (11.1)	0.173
Hypothyroidism	11 (3.8)	9 (3.2)	2 (22.2)	0.041
Values are presented as numbers and precentages (%). BMI: body mass index, CAD: coronary artery disease, HT: hypertension, DM: diabetes mellitus, SD: standard deviation, min: minimum, max: maximum				

**Table 2:** - Demographic characteristics of the pregnant women in the study.

Variables	Total	Disease severity		*P*-values
		Mild-moderate	Severe-critical	
Length of stay in ICU (days), mean±SD (min-max)	9.2±8.5 (1-26)	2.0±1.4 (1-3)	11.0±8.6 (3-26)	0.064
Having COVID-19 before pregnancy	4 (1.4)	4 (1.4)	0 (0.0)	1.000
Vaccinated	4 (1.4)	4 (1.4)	0 (0.0)	1.000
***Which vaccine?***				
Two doses of SNVC	2 (50.0)	2 (50.0)		-
Two doses of BTC	2 (50.0)	2 (50.0)		
***In which trimester?***				
First trimester	66 (22.7)	65 (23.0)	1 (11.1)	0.013
Second trimester	100 (34.4)	100 (35.5)	0 (0.0)	
Third trimester	125 (43.0)	117 (41.5)	8 (88.9)	
***Disease severity***				
Mild	245 (84.2)	245 (86.9)	0 (0.0)	-
Moderate	37 (12.7)	37 (13.1)	0 (0.0)	
Severe	3 (1.0)	0 (0.0)	3 (33.3)	
Critical	6 (2.1)	0 (0.0)	6 (66.7)	
Hospitalization	48 (16.5)	41 (14.5)	7 (77.8)	<0.001
Length of stay in hospital (days), mean±SD (min-max)	6.4±7.5 (1-35)	4.0±2.3 (1-10)	18.9±11.9 (6-35)	<0.001
Mortality	4 (1.4)	0 (0.0)	4 (44.4)	<0.001
***Hepatitis (n=256)***				
Absent	251 (98.0)	242 (98.0)	9 (100)	1.000
HbsAg positive	5 (2.0)	5 (2.0)	0 (0.0)	
Chest X-ray	14 (4.8)	6 (2.1)	8 (88.9)	<0.001
Chest X-ray finding (n=13)	11 (84.6)	3 (60.0)	8 (100)	0.128
CT	8 (2.7)	2 (0.7)	6 (66.7)	<0.001
CT finding (n=8)	8 (100)	2 (100)	6 (100)	-
Ritonavir-lopinavir	2 (0.7)	0 (0.0)	2 (22.2)	0.001
Plaquanil	0 (0.0)	282 (100)	9 (100)	-
Favipiravir	0 (0.0)	282 (100)	9 (100)	-
***Others***	17 (5.8)	11 (3.9)	6 (66.7)	<0.001
Systemic steroid	5 (1.7)	0 (0.0)	5 (55.6)	<0.001
Immune plasma	1 (0.3)	0 (0.0)	1 (11.1)	0.031
IVIG	2 (0.7)	0 (0.0)	2 (22.2)	0.001
Tocilizumab	1 (0.3)	0 (0.0)	1 (11.1)	0.031
Values are presented as numbers and precentages (%). ICU: intensive care unit, COVID-19: coronavirus disease 2019, SD: standard deviation, min: minimum, max: maximum, SNVC: Sinovac, BTC: BioNTech, HbsAg: hepatitis B surface antigen, CT: computed tomography, IVIG: intravenous immunoglobulin				

When the laboratory values were assessed, the values of white blood cells (WBC, *p*<0.001), peripheral blood neutrophils (*p*=0.001), high-sensitive C-reactive protein (Hs-CRP, *p*=0.001), aspartate aminotransferase (AST, *p*=0.001), D-dimer (*p*=0.001), ferritin (*p*<0.001), and procalcitonin (*p*<0.001) were found to be higher in the severe-critical cases than in mild-moderate cases (the highest values were assessed).

In the univariate analyses, according to binary logistic regression analysis, BMI (*p*=0.004), dyspnea (*p*<0.001), cough (*p*<0.001), maternal complication frequency (*p*=0.001), NLR (*p*<0.001), WBC (*p*=0.003), procalcitonin (*p*=0.001), Hs-CRP (*p*<0.001), D-dimer (*p*=0.029), ferritin (*p*=0,001), AST (*p*=0.006), and alanine aminotransferase (ALT; *p*=0.004) were detected as significant risk factors (Table 3). In the multivariate analysis, only procalcitonin was determined to be a significant risk factor (*p*=0.032, Table 3).

**Table 3:** - Factors determining severe critical illness univariate and multivariate logistic regression analysis.

Variables	*P*-values	OR	95% CI
Number of pregnancies	0.457	1.149	0.796-1.658
BMI (kg/m^2^)	0.004	7.143	1.842-27.692
First trimester	0.380		
Second trimester	0.997	0.000	0.000
Third trimester	0.164	4.444	0.544-36.325
Dyspnea	<0.001	45.714	5.573-375.011
Cough	<0.001	69.793	8.427-578.018
Maternal complication	0.001	24.937	3.886-160.026
NLR	<0.001	1.169	1.081-1.265
WBC (µL)	0.003	1.000	1.000-1.001
Procalcitonin(ng/ml)	0.001	49.808	4.748-522.534
CRP (mg/dl)	<0.001	1.040	1.021-1.060
D-dimer (ng/ml)	0.029	1.000	1.000-1.001
Ferritin (ng/ml)	0.001	1.018	1.007-1.028
AST (U/L)	0.006	1.015	1.004-1.026
ALT (U/L)	0.004	1.016	1.005-1.026
***Multivariate logistic regression analysis forward method***			
Procalcitonin	0.032	15.955	1.261-201.927
BMI: body mass index, NLR: neutrophil lymphocyte ratio, WBC: white blood cell, CRP: C-reactive protein, AST: aspartate aminotransferase, ALT: alanine aminotransferase, OR: odds ratio			

No statistically significant difference was determined between patients with and without asthma in respect of the rates of severe-critical disease (*p*=1.000), hospitalization (*p*=0.485), admission to ICU (*p*=1.000), and mortality (*p*=1.000).

The cases in the third trimester were determined to have higher rates of severe-critical disease (*p*=0.027), hospitalization (*p*=0.008), and mortality (*p*=0.048) than the patients in other trimesters.

The number of previous pregnancies was determined to be positively correlated with the severity of the disease, Hs-CRP, AST, and ferritin, and negatively correlated with peripheral blood eosinophil values. The trimester in which the COVID-19 infection was diagnosed showed a positive correlation with disease severity, WBC, neutrophil, Hs-CRP, D-dimer, and creatinine, and a negative correlation with hemoglobin. The severity of the disease was positively correlated with Hs-CRP, AST, D-dimer, ferritin, and procalcitonin, and negatively correlated with hemoglobin and peripheral blood eosinophil values (Table 4).

**Table 4:** - Corrrelations between the number of pregnancies and the severity of the disease.

Variables	Number of pregnancies		In which trimester?		Disease severity	
	r	*P*-values	r	*P*-values	r	*P*-values
Disease severity	0.140	0.017	0.171	0.003		
Hemoglobin (g/dl)	-0.048	0.592	-0.213	0.016	-0.221	0.012
WBC (µL)	-0.143	0.108	0.242	0.006	0.160	0.073
Neutrophil (µL)	-0.073	0.413	0.283	0.001	0.154	0.083
Lymphocyte (µL)	-0.158	0.077	-0.084	0.350	-0.142	0.112
CRP (mg/dl)	0.252	0.006	0.194	0.034	0.429	<0.001
Eosinophil (µL)	-0.215	0.015	0.016	0.863	-0.192	0.032
AST (U/L)	0.196	0.032	0.116	0.207	0.311	0.001
ALT (U/L)	0.148	0.104	0.002	0.985	0.138	0.131
D-dimer (ng/ml)	0.161	0.204	0.494	<0.001	0.489	<0.001
Ferritin (ng/ml)	0.232	0.039	0.123	0.281	0.429	<0.001
Creatinine (mg/dl)	0.010	0.916	0.187	0.042	0.127	0.167
Procalcitonin (ng/ml)	-0.006	0.962	0.173	0.157	0.544	<0.001
WBC: white blood cell, CRP: C-reactive protein, AST: aspartate aminotransferase, ALT: alanine aminotransferase						

**Figure 2:**
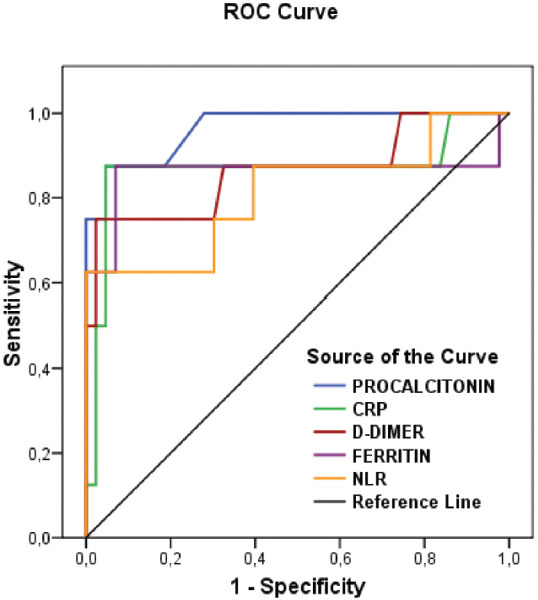
- The results of receiver operating characteristic curve analysis for C-reactive protein, D-dimer, ferritin, and neutrophil/lymphocyte ratio. ROC: receiver operating characteristic curve, CRP: C-reactive protein, NLR: neutrophil/lymphocyte ratio

For severe-critical diseases, the optimal cut-off value of procalcitonin was calculated as 0.425 (AUC=0.965, 95% CI: [0.907-1.022]) with 87.5% sensitivity and 95% specificity. The cut-off value for CRP was calculated as 105.5 (AUC=0.868, 95% CI: [0.675-1.061]) with 87.5% sensitivity, and 95.3% specificity. For D-dimer, the cut-off value was calculated as 2000 (AUC=0.863, 95% CI: [0.688-1.039]) with 75% sensitivity and 79.1% specificity. The cut-off value for ferritin was determined to be 161.5 (AUC=0.860, 95% CI: [0.639-1.082]) with 87.5% sensitivity and 93% specificity, and for NLR, the cut-off value was calculated as 5.79 (AUC=0.811, 95% CI: [0.611-1.011]) with 75% sensitivity and 69.8% specificity (Figure 2).

## Discussion

This study with evaluations from a single province in the Southeast of Turkey with a population of 620,00011 contributes significant information to the medical literature. The cases in the third trimester of pregnancy were observed to experience more severe-critical disease than the women in the earlier periods of pregnancy. It was also determined that the severe-critical cases and the cases which resulted in mortality were mostly seen in the last period of the study, and the 4 patients with mortality had not been vaccinated against the disease.

Although more than 90% of pregnant women with COVID-19 infection recover without hospitalization, a rapid clinical deterioration can be seen in some cases. Some studies have reported that symptomatic pregnant patients were at a higher risk of severe disease and mortality compared to symptomatic non-pregnant women of reproductive age.^12,13^ In another study, it was reported that pregnant women with COVID-19 positivity were at higher risk of mortality than those without COVID-19, but not at higher risk than non-pregnant women of reproductive age with COVID-19 infection.^14^ In a study covering the first 5 months of the pandemic, in which 75 hospitalized pregnant patients were evaluated, the admission rate to the ICU was found to be 2.7% and the clinical course of the disease in these patients was similar to that of the general population.^15^ In a meta-analysis, the mortality rate was 1% in SARS-CoV-2 infected pregnant women.^16^ In the present study, covering the first 18 months of the pandemic, the rate of hospitalization was 16.5%, the rate of admission to the ICU was 3.1%, and the rate of mortality was 1.3%. A study from the United States of America reported that 27% of SARS-CoV-2 infected pregnant women had mild disease (26%), severe, and critical disease (5%).^17^ In the current study, these rates for pregnant women with confirrmed COVID-19 infection were 84.2% for mild, 12.7% for moderate, 1% for severe, and 2.1% for critical disease, and the high rate of mild disease course was thought to be related to the screening of all patients, including both outpatients and those who were hospitalized.

Age of ≥35 years, obesity, and pre-existing comorbidities (hypertension, diabetes mellitus, and others) have been determined as risk factors for severe COVID-19 disease and mortality.^12,13^ In the current study, high BMI values (*p*=0.014), the presence of dyspnea (*p*<0.001), cough (*p*<0.001), and comorbidities (*p*=0.041) were detected at higher rates in severe-critical cases. Furthermore, a higher rate of hypothyroidism was found in severe-critical cases (*p*=0.041) similar to the findings in another study.^18^ The clinical effects of asthma on COVID-19 remain unclarified. It has been suggested that an increased vulnerability to the SARS-CoV-2 virus and a more severe disease course are due to the higher risk of exacerbations by viral infections.^19^ In another study, it was suggested that accumulated eosinophils and type II inflammation cytokines (IL-4, IL-5, and IL-13) were protective.^19,20^ In the present study, no significant differences were detected in the disease severity (*p*=1.000), hospitalization (*p*=0.485), admission to ICUs (*p*=1.000), and mortality (*p*=1.000) between the patients with and without asthma.

The findings of this study demonstrated that the values of WBC (*p*<0.001), neutrophil (*p*=0.001), Hs-CRP (*p*=0.001), AST (*p*=0.001), D-dimer (*p*=0.001), ferritin (*p*<0.001), and procalcitonin (*p*<0.001) were higher in severe-critical cases than in mild-moderate cases. These findings are not specific to pregnant women but are the same as in the general population with COVID-19 infection.^21^ It was also shown in the univariate analysis that obesity, the symptoms of dyspnea and cough, the parameters of NLR, WBC, procalcitonin, Hs-CRP, D-Dimer, ferritin, AST, and ALT were significant risk factors for severe-critical disease.

For pregnant patients, the type and timing of delivery should be decided according to the severity of COVID-19, the presence of comorbidities and obstetric indications. In a study evaluating 230 pregnant patients, the rates of caesarean delivery were higher than vaginal delivery and these were mostly premature births.^22^ In another study, a higher rate of caesarean delivery was reported but there was no supporting evidence as the COVID-19 infection mostly did not threaten the maternal health status.^23^ In a meta-analysis covering 61 studies, 790 SARS-CoV-2 infected pregnant patients and 548 newborns were analysed. The rate of caesarean delivery was 72% and premature births was 23%.^24^ In the current study, caesarean delivery was determined at the rate of 40.2% and premature births at 17.5%. Importantly, cesarean delivery was more common in the severe-critical cases, most of which were diagnosed in the third trimester.

According to the COVID-19 management guidelines of the Turkish Health Ministry, no treatment was recommended for asymptomatic pregnant women.^25^ Even if there was no contra-indication for screening lungs with CT, it was also recommended to avoid CT as much as possible.^26^ Therefore, the screening rates of CT and X-ray were found to be higher in the severe-critical cases in this study. According to the same guidelines, follow-up without treatment was recommended for non-severe SARS-CoV-2 infected pregnant women.^25^ Thus, only the severe-critical cases in this study were treated with appropriate treatment options.

The rate of spontaneous abortion was estimated to be 10% in women aged 25-29 years, and approximately 57% in those aged ≥45 years according to a national prospective cohort study.^27^ In a recent study from Turkey, it was stated that COVID-19 affected pregnancy and increased the rates of maternal mortality, stillbirth, and abortus.^28^ In the current study, 13 (4.5%) pregnancies resulted in spontaneous abortion. Consistent with the findings of some other studies, COVID-19 infection was not seen to increase the risk of spontaneous abortion.^26^

A previous review,^18^ stated that asymptomatic and mild cases are more commonly seen in the third trimester, whereas another review claimed that the third trimester was the most vulnerable period of the pregnancy for COVID-19 infection.^29^ In the present study, 8 (88.9%) of the 9 severe-critical cases were in the third trimester and the remaining one (11.1%) patient was in the first trimester. In addition to a higher rate of severe-critical disease (*p*=0.027), higher rates of hospitalization (*p*=0.008), and mortality (*p*=0.048) were determined in patients in the third trimester than in those at the earlier stages of pregnancy.

Considering that pregnant women are generally under the age of 40 years, these patients had the right to take a COVID-19 vaccine in June 2021 in Turkey. After this date, 102 (35%) pregnant women were diagnosed with COVID-19 infection and only 4 patients had a COVID-19 vaccination. These vaccinated patients were diagnosed in August 2021 and recovered following a mild clinical course. The 3 patients in this study who developed mortality were diagnosed with COVID-19 in August (the recent period) and none had received a COVID-19 vaccination. Therefore, it might be suggested that new variants of the virus showed a deleterious clinical effect.

### Study limitations

First, non-pregnant women could have been recruited into the study as a control group. Second, due to the retrospective design of the study, it was not possible to identify clinical changes in the data in a prospective manner.

In conclusion, the most interesting finding of the study was the positive correlation between the number of pregnancies and the severity of COVID-19. It was also seen that the severity of the disease worsened mostly in the recent period of the pandemic, possibly due to new variants of the virus. Physicians should be aware of the increased risks for pregnant women with obesity, hypothyroidism, or in the third trimester. Considering the clinical outcomes of the vaccinated pregnant women in this study, COVID-19 vaccination seems to be the only option to avoid severe disease.
